# An effective and effecient peptide binding prediction approach for a broad set of HLA-DR molecules based on ordered weighted averaging of binding pocket profiles

**DOI:** 10.1186/1477-5956-11-S1-S15

**Published:** 2013-11-07

**Authors:** Wen-Jun Shen, Shaohong Zhang, Hau-San Wong

**Affiliations:** 1 Department of Computer Science, City University of Hong Kong, Kowloon, Hong Kong; 2 Department of Computer Science, Guangzhou University, Guangzhou, P.R. China

## Abstract

**Background:**

The immune system must detect a wide variety of microbial pathogens, such as viruses, bacteria, fungi and parasitic worms, to protect the host against disease. Antigenic peptides displayed by MHC II (class II Major Histocompatibility Complex) molecules is a pivotal process to activate CD4+ T_H _cells (Helper T cells). The activated T_H _cells can differentiate into effector cells which assist various cells in activating against pathogen invasion. Each MHC locus encodes a great number of allele variants. Yet this limited number of MHC molecules are required to display enormous number of antigenic peptides. Since the peptide binding measurements of MHC molecules by biochemical experiments are expensive, only a few of the MHC molecules have suffecient measured peptides. To perform accurate binding prediction for those MHC alleles without suffecient measured peptides, a number of computational algorithms were proposed in the last decades.

**Results:**

Here, we propose a new MHC II binding prediction approach, OWA-PSSM, which is a significantly extended version of a well known method called TEPITOPE. The TEPITOPE method is able to perform prediction for only 50 MHC alleles, while OWA-PSSM is able to perform prediction for much more, up to 879 HLA-DR molecules. We evaluate the method on five benchmark datasets. The method is demonstrated to be the best one in identifying binding cores compared with several other popular state-of-the-art approaches. Meanwhile, the method performs comparably to the TEPITOPE and NetMHCIIpan2.0 approaches in identifying HLA-DR epitopes and ligands, and it performs significantly better than TEPITOPEpan in the identification of HLA-DR ligands and MultiRTA in identifying HLA-DR T cell epitopes.

**Conclusions:**

The proposed approach OWA-PSSM is fast and robust in identifying ligands, epitopes and binding cores for up to 879 MHC II molecules.

## Introduction

The immune system must detect a wide variety of microbial pathogens, such as viruses, bacteria, fungi and parasitic worms, to protect the host against disease. Antigenic peptides displayed by MHC II (class II Major Histocompatibility Complex) molecules is a pivotal process to activate CD4+ T_H _cells (Helper T cells). The activated T_H _cells can differentiate into effector cells which assist various cells in activating against pathogen invasion [1]. MHC I and II are the two main classes of MHC. MHC I molecules exist in all nucleated cells. CD8+ T cytotoxic cells only recognize antigenic peptides which are displayed by MHC I from cytosol to the surface of cells and eliminate the infected cells. On the other hand, MHC II molecules are normally found only in antigen-presenting cells (APCs). T_H _cells only recognize those foreign peptides that are displayed by MHC II from endocytosed proteins to the surface of APCs and then produce a large number of cytokineses to activate various cells to defend invasion [2,3].

The structures of MHC I and II are slightly different on the binding grooves. MHC I molecules have conserved residues which bind to the terminal residues of antigenic peptides, so they form close grooves. On the other hand, these kinds of conserved residues do not exist in the MHC II molecules, which form open grooves. Hence MHC II can accommodate longer peptides than MHC I, which results in increased diffeculty in performing binding prediction for MHC II [4-6].

The HLA (Human Leukocyte Antigen, MHC in humans) II molecules are encoded by the DP, DQ and DR loci. Each MHC locus encodes a great number of allele variants. Yet this limited number of MHC molecules are required to display enormous number of antigenic peptides. Each specific MHC molecule can bind to a great number of different peptides, and certain peptides can bind to several MHC molecules. Since the peptide binding measurements of MHC molecules by biochemical experiments are expensive, only a few of the MHC molecules have suffecient measured peptides. In [7], it is mentioned that in order to accurately describe the binding motif of MHC II, at least 100 to 200 measured peptides are required. To perform accurate binding prediction for those MHC alleles without suffecient measured peptides, a number of computational algorithms (referred to as pan-specific methods) were proposed in the last decade [8,9].

The TEPITOPE [10] method is the pioneering and most popular pan-specific approach for MHC II binding prediction. Its basic idea is if two HLA-DR alleles have identical pseudo sequence (The pseudo sequence is composed of several amino acids.) in the same pocket, they will share the same quantitative profile (The pocket profile measures the binding strength between a given pocket with the twenty basic amino acids.). The MHCIIMulti [11] method enables prediction of more than 500 HLA-DR molecules by using multiple instance learning. The NetMHCIIpan [12] method first transforms each DRB allele into a 21 amino acids pseudo-sequence, and uses the SMM-align [7] method to identify the binding cores and peptide flanking residues, next trains the model using an artificial neural network learning algorithm. The MultiRTA [13] method, which can perform prediction for both HLA-DR and HLA-DP molecules, calculates the binding affenity of a peptide by thermodynamic averaging over the binding affenities of all registers, and introduces a regularization constraint to avoid overfitting. The NetMHCIIpan-2.0 [14] method is a synthesis of NN-align [15], NetMHCpan and NetMHCIIpan. MULTIPRED2 [16] can perform prediction for 1077 HLAI and HLA-II alleles and 26 HLA supertypes. It can be regarded as a combination of the MULTIPRED, PEPVAC, NetMHCpan and NetMHCIIpan methods. The TEPITOPEpan [9] method, which builds on the TEPITOPE and PickPocket [17] methods, enables prediction for more than 700 HLA-DR molecules.

Here, we propose a new MHC II binding prediction method, which we call OWA-PSSM. A preliminary study of a special case of this framework was first conducted in [18]. This method is a significantly extended version of the TEPITOPE method. Through introducing the ordered weighted averaging (OWA) weights [19,20], we develop a novel weighting scheme for those pocket profiles generated by TEPITOPE. Specifically, the gamma probability density function (PDF) [21] is employed to generate the OWA weights. The gamma PDF is a generalization of the exponential density function, and have close relationship with a number of continuous distributions. In our experiments, we will evaluate the performance of OWA-PSSM through comparing with four other popular state-of-the-art or recently proposed pan-specific methods, TEPITOPE, MultiRTA, NetMHCIIpan2.0 and TEPITOPEpan.

## Materials and methods

We retrieved all HLA-DRB (HLA-DR *β *chain) protein sequences from the FTP site (ftp://ftp.ebi.ac.uk/pub/databases/ipd/imgt/hla/) provided by the IMGT/HLA database.

Five independent benchmark datasets are employed to evaluate the performance of OWA-PSSM through comparing with the TEPITOPE, MultiRTA, NetMHCIIpan2.0 and TEPITOPEpan methods.

## Data sets

### Generation of 879 DRB alleles

A set  D of 879 DRB alleles are prepared as follows. We retrieved all DRB protein sequences from the FTP site provided by the IMGT/HLA database. Each DRB allele has an offecial name assigned by the WHO Nomenclature Committee for Factors of the HLA System. The first four digits coming after the gene name are used to distinguish different alleles. Hence for those alleles without difference until the fifth digit, we kept only the first one of them by sorting their offecial names in ascending order. Meanwhile, for each allele, we only considered the residues whose IMGT assigned residue indices range from 9 to 86 as they cover all pocket residues employed in TEPITOPE. Those alleles with absent amino acids in this range are omitted. The final step is to exclude non-expressed alleles and those alleles whose amino acid at residue 86 is neither glycine nor valine.

### SMM-align DRB binding dataset

The substitution matrix and the parameters of the gamma PDF are determined by using the same dataset [7] as the TEPITOPEpan method. Hence these two methods will be compared in a more compatible way.

### MHCBench DRB1*0401 binding dataset

The MHCBench server [22] provides eight benchmark datasets to evaluate MHC binding prediction methods. It is a popular benchmark to evaluate the performance of new methods by comparing with previously developed algorithms.

### NetMHCIIpan-2.0 HLA-DR ligands

A large dataset studied in [14] consisting of 1164 HLA-DR ligands and 28 DRB alleles is evaluated.

### NetMHCIIpan-2.0 HLA-DR T cell epitopes

Another large dataset studied in [14] consisting of 42 DRB alleles and 1325 epitopes is adopted to perform further evaluation.

### X-ray crystallographic structures of pMHC II complex

The last dataset contains 41 X-ray crystallographic structures of pMHC II complexes (see Table 1). These 41 X-ray structures were retrieved from the PDB database [23]. For these 41 X-ray structures, each one contains an HLA-DR/peptide binding complex. The binding cores were directly obtained from the IMGT/3Dstructure database [24]. To the best of our knowledge, this dataset is the largest and most complete that has ever been studied for the prediction of MHC II binding cores.

**Table 1:** X-ray crystallographic structures of pMHC II binding complexes.

PDB ID	DRB Allele	Peptide Sequence	Core
4E41	DRB1*0101	GELIGILNAAKVPAD	IGILNAAKV
1A6A	DRB1*0301	PVSKMRMATPLLMQA	MRMATPLLM
1AQD	DRB1*0101	VGSDWRFLRGYHQYA	WRFLRGYHQ
1BX2	DRB1*1501	ENPVVHFFKNIVTPR	VHFFKNIVT
1DLH	DRB1*0101	PKYVKQNTLKLAT	YVKQNTLKL
1FV1	DRB5*0101	NPVVHFFKNIVTPRTPPPSQ	FKNIVTPRT
1FYT	DRB1*0101	PKYVKQNTLKLAT	YVKQNTLKL
1H15	DRB5*0101	GGVYHFVKKHVHES	YHFVKKHVH
1HQR	DRB5*0101	VHFFKNIVTPRTP	FKNIVTPRT
1HXY	DRB1*0101	PKYVKQNTLKLAT	YVKQNTLKL
1J8H	DRB1*0401	PKYVKQNTLKLAT	YVKQNTLKL
1JWM	DRB1*0101	PKYVKQNTLKLAT	YVKQNTLKL
1JWS	DRB1*0101	PKYVKQNTLKLAT	YVKQNTLKL
1JWU	DRB1*0101	PKYVKQNTLKLAT	YVKQNTLKL
1KG0	DRB1*0101	PKYVKQNTLKLAT	YVKQNTLKL
1KLG	DRB1*0101	GELIGILNAAKVPAD	IGILNAAKV
1KLU	DRB1*0101	GELIGTLNAAKVPAD	IGTLNAAKV
1LO5	DRB1*0101	PKYVKQNTLKLAT	YVKQNTLKL
1PYW	DRB1*0101	XFVKQNAAAL	FVKQNAAAL
1R5I	DRB1*0101	PKYVKQNTLKLAT	YVKQNTLKL
1SJE	DRB1*0101	PEVIPMFSALSEGATP	VIPMFSALS
1SJH	DRB1*0101	PEVIPMFSALSEG	VIPMFSALS
1T5W	DRB1*0101	AAYSDQATPLLLSPR	YSDQATPLL
1T5X	DRB1*0101	AAYSDQATPLLLSPR	YSDQATPLL
1YMM	DRB1*1501	ENPVVHFFKNIVTPRGGSGGGGG	VHFFKNIVT
1ZGL	DRB5*0101	VHFFKNIVTPRTPGG	FKNIVTPRT
2FSE	DRB1*0101	AGFKGEQGPKGEPG	FKGEQGPKG
2G9H	DRB1*0101	PKYVKQNTLKLAT	YVKQNTLKL
2IAM	DRB1*0101	GELIGILNAAKVPAD	IGILNAAKV
2IAN	DRB1*0101	GELIGTLNAAKVPAD	IGTLNAAKV
2ICW	DRB1*0101	PKYVKQNTLKLAT	YVKQNTLKL
2IPK	DRB1*0101	XPKWVKQNTLKLAT	WVKQNTLKL
2OJE	DRB1*0101	PKYVKQNTLKLAT	YVKQNTLKL
2Q6W	DRB3*0101	AWRSDEALPLGS	WRSDEALPL
2SEB	DRB1*0401	AYMRADAAAGGA	MRADAAAGG
3C5J	DRB3*0301	QVIILNHPGQISA	IILNHPGQI
3L6F	DRB1*0101	APPAYEKLSAEQSPP	YEKLSAEQS
3PDO	DRB1*0101	KPVSKMRMATPLLMQALPM	MRMATPLLM
3PGD	DRB1*0101	KMRMATPLLMQALPM	MRMATPLLM
3S4S	DRB1*0101	PKYVKQNTLKLAT	YVKQNTLKL
3S5L	DRB1*0101	PKYVKQNTLKLAT	YVKQNTLKL

## Methods

The proposed OWA-PSSM approach is introduced in the following subsections. The OWA-PSSM method is designed based on the PSSM (Position Specific Scoring Matrix) which is a popular technique in the prediction of MHC binding [9,10,17,25-28]. In general, the lengths of MHC II binding cores are nine amino acids. Every position at the binding core is related to a specific pocket. The PSSM is employed to specify the binding strengths between twenty basic amino acids with these nine pockets, such that the binding specificities of HLA-DR molecules could be quantified.

For MHC II molecules, there are five anchor sites (sites 1, 4, 6, 7 and 9) at the binding core. These five anchor sites govern the binding strength of peptides with MHC II molecules [3]. The OWA weights are employed to define profiles for anchor pockets 4, 6, 7 and 9. For the remaining pockets, including the anchor pocket 1 and four non-anchor pockets 2, 3, 5 and 8, we adopt the same strategy as TEPITOPE to specify their quantitative profiles.

### Generation of profiles for pockets 4 6 7 9

Here, the pocket pseudo-sequences and the associated profiles generated by TEPITOPE are referred to as raw pocket pseudo-sequences and raw pocket profiles, respectively. These raw pseudo-sequences are composed of several amino acids whose associated residue indices are given in Table 2. Eleven representative HLA-DR alleles are adopted by TEPITOPE to specify the different profiles for anchor pockets 4, 6, 7 and 9. These eleven alleles are DRB1*0101, DRB1*0301, DRB1*0401, DRB1*0402, DRB1*0404, DRB1*0701, DRB1*0801, DRB1*1101, DRB1*1302, DRB1*1501 and DRB5*0101. If two alleles have identical pseudosequences in the same pocket, they will have identical profiles. For a given pocket (pocket 4, 6, 7 or 9), we collect all the different raw pocket pseudo-sequences into a set $R^{x}$, $R^{x}=\left\{r_{1},\;r_{2},\ldots ,\;r_{m}\right\}$, *|r_i_| *= *n*, where *i *= 1, 2, ..., *m*, *x *∈ {4, 6, 7, 9}, *m *is the number of unique pseudo-sequences, and *n *is the number of amino acids contained in a pseudo-sequence. Meanwhile, we collect all the different raw profiles into a set $P^{x}$, $P^{x}=\left\{p_{1},\;p_{2},\ldots ,\;p_{m}\right\}$ and *|p_i_| *= 20, *i *= 1, 2, ..., *m*. There is a one-to-one correspondence between *p_i _*and *r_i_*. By using a substitution matrix, every raw pseudo-sequence is represented as a 20*n*-dimensional real vector, and is referred to as raw encoded pseudo-sequence collected into a set $V^{x}$, $V^{x}=\left\{v_{1},\;v_{2},\ldots ,\;v_{m}\right\}$.

**Table 2:** DRB Pocket Residue Indices.

Pocket	Pocket residue indices
P1	86
P2	-
P3	-
P4	13, 70, 71, 74, 78
P5	-
P6	11
P7	28, 30, 47, 61, 67, 71
P8	-
P9	9, 37, 57, 60, 61

Next the radial basis function (RBF) is employed to measure the similarity between the encoded pseudo-sequences of a predicted allele *a *and a raw encoded pseudo-sequence.

(1) $$K\left(v_{a},v_{i}\right)=\text{exp}\left(-\frac{1}{2}{∥v_{a}-v_{i}∥}^{2}\right),v_{i}\in V^{x},$$

where *v_a _*is the encoded pseudo-sequence for a predicted allele *a*. The pseudo-sequence for allele *a *is generated by using the pocket residue indices in Table 2.

Obviously, 0 *< K*(*v_a_, v_i_*) ≤ 1 and *K*(*v_a_, v_i_*) = 1 if and only if *v_a _*= *v_i_*.

**Figure 1:**
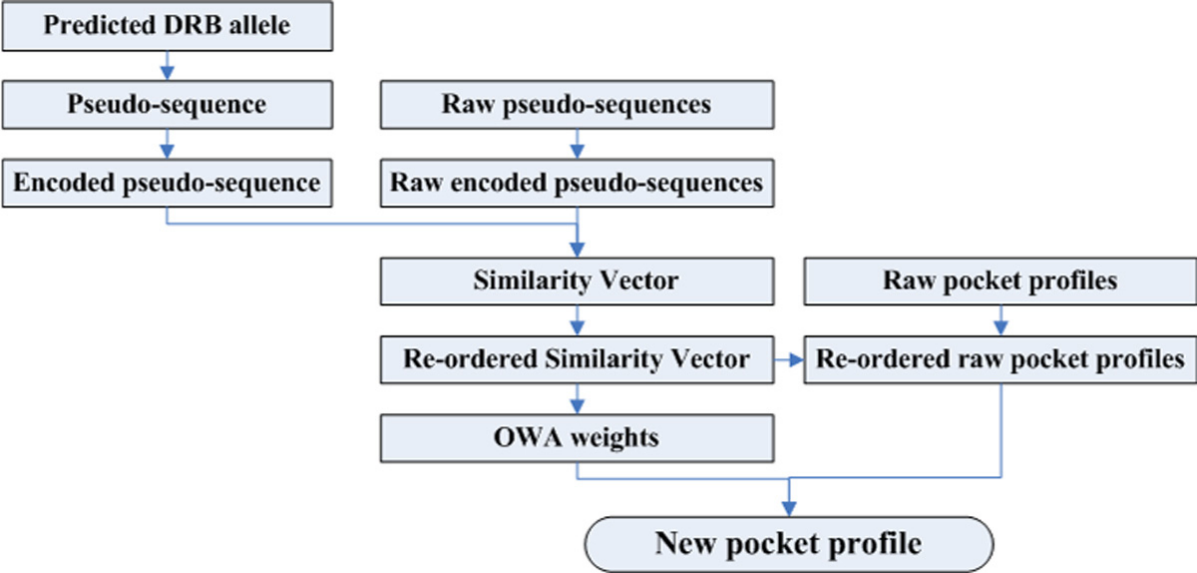
**Schematic illustration of profile generation for pocket 4/6/7/9**.

After these similarity values are sorted in descending order, a specific ordered position would be associated with an OWA weight. A new pocket profile is generated as a weighted average over *m *raw pocket profiles in $P^{x}$. A schematic illustration of the generation of a new profile is shown in Figure 1[18].

Next we use the gamma distribution to generate OWA weights. The PDF of a gamma distribution is defined by:

$$g\left(x;k,\theta \right)=\frac{1}{\theta^{k}}\frac{1}{\Gamma \left(k\right)}x^{k-1}e^{-\frac{x}{\theta }}$$

for *x >*0 and *k, θ >*0. Γ() denotes the gamma function.

The gamma distribution is specified by its shape and scale parameters: *k *and *θ*. When *k *≤ 1, the density function is decreasing while *k >*1, it is unimodal and the mode occurs at (*k - *1)*θ*. If *k *= 1 and *θ *= *µ*, then the gamma distribution becomes the exponential distribution *X ~ *exp(1*/µ*).

The OWA weight distribution is generated by discretizing the gamma PDF as follows:

(2) $$G\left(X=i\right)=\frac{1}{\theta^{k}}\frac{1}{\Gamma \left(k\right)}i^{k-1}e^{-\frac{i}{\theta }},i=1,2,\ldots ,m.$$

where *m *is the dimension of the OWA weights, *k *and *θ *are the shape and scale parameters respectively.

After normalizing, the OWA weights are defined by:

(3) $$P\left(X=i\right)=\frac{G\left(X=i\right)}{\sum_{k=1}^{m}G\left(X=k\right)},i=1,2,\ldots ,m.$$

Let *w_i _*= *P *(*X *= *i*), and these weights satisfy the following constraints: $\sum_{i=1}^{m}w_{i}=\text{1};w_{i}\in \left(0,\text{1}\right).$

Given a predicted DRB allele *a*, let *K_a _*= (*k*_*a*1_, *k*_*a*2_, ..., *k_am_*), where *k_ai _*= *K*(*v_a_, v_i_*), $v_{i}\in V^{x}$, and the associated raw pocket profiles are $P^{x}=\left\{p_{1},\;p_{2},\ldots ,\;p_{m}\right\}$. The elements of *K_a _*are sorted in descending order, and the re-ordered vector of *K_a _*is denoted as ${\tilde{K}}_{a}=\left({\tilde{K}}_{a1},{\tilde{K}}_{a2},\ldots ,{\tilde{K}}_{am}\right)$. The corresponding OWA weighting vector is denoted as *W *, *W *= (*w*_1_, *w*_2_, ..., *w_m_*). We denote the pocket profiles associated with the re-ordered vector ${\tilde{K}}_{a}$ as ${\tilde{P}}^{x}$, ${\tilde{P}}^{x}=\left\{{\tilde{p}}_{1},{\tilde{p}}_{2},\ldots ,{\tilde{p}}_{m}\right\}$. Hence we define the pocket profile for allele *a *by:

(4) $${\tilde{p}}_{a}^{x}=w_{1}{\tilde{p}}_{1}+w_{2}{\tilde{p}}_{2}+\ldots +w_{m}{\tilde{p}}_{m},$$

where *x *∈ {4, 6, 7, 9}.

In particular,

(5) $${\tilde{p}}_{a}^{x}=\left\{\begin{array}{cc} w_{1}{\tilde{p}}_{1}+w_{2}{\tilde{p}}_{2}+\ldots +w_{11}{\tilde{p}}_{11}, & x=4,7,\\ w_{1}{\tilde{p}}_{1}+w_{2}{\tilde{p}}_{2}+\ldots +w_{6}{\tilde{p}}_{6}, & x=6,9. \end{array}\right)$$

Essentially, through measuring the similarity between the encoded pseudo-sequence of *a *with *m *raw encoded pseudo-sequences in the same pocket *x*, the smaller the *k_ai _*the higher weight assigned to *p_i_*, $p_{i}\in P^{x}$.

The new profile weighting approach developed in this work is inspired by the OWA operator [19]. We generate profiles of pockets 4, 6, 7 and 9 for any allele in the set  D by using the OWA weights derived from the gamma PDF.

### Generation of profiles for pockets 1 2 3 5 8

For the remaining pockets, including the anchor pocket 1 and four non-anchor pockets 2, 3, 5 and 8, we adopt the same strategy as TEPITOPE to specify quantitative profiles. Accurately quantifying pocket 1 is essential for the identification of binding cores, and this pocket is mainly characterized by the 86th residue of DRB protein sequences. For all the alleles in the set  D, the amino acid at the 86th residue is either glycine or valine. For the alleles with glycine at the 86th residue, the profile of pocket 1 is assigned to be -1 for aliphatic amino acids (Ile, Leu, Met, Val) and 0 for aromatic amino acids (Phe, Trp, Tyr). However, if this residue is valine, the profile is set to 0 for aliphatic and -1 for aromatic. Other amino acids are set to -999 [29]. This reflects that the binding cores of MHC II prefer aliphatic and aromatic amino acids at position 1. For the non-anchor pockets 2, 3, 5 and 8, their contributions to the pMHC binding is minimal. Hence we assign identical profiles to all alleles for pockets 2 and 3 and zero vectors for pockets 5 and 8 [29], respectively. Given an allele a∈D, the pocket profiles are denoted as ${\tilde{p}}_{a}^{x}$, *x *= 1, 2, 3, 5, 8.

### Position specific scoring matrices

For a given allele a∈D, the quantitative profiles for nine pockets are defined by ${\tilde{P}}_{a}=\left\{{\tilde{p}}_{a}^{1},{\tilde{p}}_{a}^{2},\ldots ,{\tilde{p}}_{a}^{9}\right\}$ Then the PSSM is defined by assembling nine pocket profiles together as

$$PSSM_{a}=\left[{\tilde{p}}_{a}^{1},{\tilde{p}}_{a}^{2},\ldots ,{\tilde{p}}_{a}^{9}\right].$$

The PSSM is a 20 by 9 matrix whose columns correspond to nine pockets and rows correspond to twenty basic amino acids.

### Prediction measure and statistical tests

The AUC (Area Under the receiver operating characteristic Curve) is employed to measure the prediction performance, which is 1 for perfect prediction and 0.5 for random prediction.

A paired *t *test is used for statistical comparison, and the AUC score comparison result is considered to be statistically significant if p is less than 0.05.

## Results

The substitution matrix and the parameters *k *and *θ *of the gamma PDF are determined by using the dataset described in [7], which contains 14 HLA-DR alleles. The MHCBench, NetMHCIIpan-2.0 HLA-DR ligand and T cell epitope datasets were then used to extensively evaluate the performance of OWA-PSSM through comparing with TEPITOPE, MultiRTA, NetMHCIIpan2.0 and TEPITOPEpan. Furthermore, 41 X-ray crystallographic structures of pMHC II complexes were employed to evaluate the prediction quality of OWA-PSSM on identifying binding cores.

### Determination of the substitution matrix and the gamma distribution parameters

A substitution matrix is used to encode an amino acid into a 20-dimensional real-valued vector. We could then apply the Gaussian kernel to compute similarities between encoded pseudo-sequences. The BLOSUM50 and BLOSUM62 [30] matrices are two important substitution matrices for MHC/peptide binding prediction [9,12,17]. Here, 59 symmetric substitution matrices are tested.

The gamma probability density function is discretized to generate OWA weights, with those values controlled by the shape and scale parameters.

Experiments were performed to explore the effect on MHC II prediction through varying the substitution matrix and gamma distribution parameters *k *and *θ*. We used the SMM-align dataset consisting of 14 HLADR alleles and 4603 peptides to determine the parameters. We tested 59 symmetric substitution matrices obtained from the AAindex database [31] whose data are collected from published literature. Optimal shape and scale parameters were chosen from set {0.1, 0.2, ..., 0.9} ∪ {1, 2, ..., 30}. We find that the chemical similarity substitution matrix [32] with shape parameter *k *= 0.2, *θ *= 0.6 performs best. The experiment results show that the shape parameter *k *≤ 1 performs better than *k *> 1. The scale parameter measures the steepness of the OWA distribution, and the OWA distribution becomes steeper when *θ *is smaller. And thus in the following the chemical similarity matrix is used to encode the pocket pseudo-sequences, and the OWA weights applied to define profiles for 879 DRB alleles in the set  D are determined by the discretization of the gamma distribution with shape parameter 0.2 and scale parameter 0.6.

The prediction performance for the SMM-align dataset is shown in Table 3. The performance of OWAPSSM is better than TEPITOPE and TEPITOPEpan. The average AUC scores over these 14 DRB alleles are 0.747 and 0.732 for the OWA-PSSM and TEPITOPEpan, respectively. The performance of OWA-PSSM, TEPITOPE and TEPITOPEpan are comparable for the alleles predictable by TEPITOPE. However, for those alleles not predictable by TEPITOPE, the average AUC scores are 0.776 and 0.711 for OWA-PSSM and TEPITOPEpan, respectively. OWA-PSSM outperforms TEPITOPEpan in all alleles not predictable by TEPITOPE.

**Table 3:** The performance of OWA-PSSM, TEPITOPE and TEPITOPEpan on the SMM-align dataset in terms of AUC.

Allele	# peptides	TEPITOPE	TEPITOPEpan	OWA-PSSM
DRB1*0101	1203	0.647	0.648	0.645
DRB1*0301	474	0.733	0.739	0.731
DRB1*0401	457	0.754	0.770	0.756
DRB1*0404	168	0.829	0.832	0.830
DRB1*0405	171	0.789	0.785	0.789
DRB1*0701	310	0.768	0.768	0.771
DRB1*0802	174	0.769	0.774	0.784
DRB1*0901	117		0.686	0.731
DRB1*1101	359	0.709	0.700	0.715
DRB1*1302	179	0.721	0.728	0.727
DRB1*1501	365	0.725	0.727	0.731
DRB3*0101	102		0.724	0.869
DRB4*0101	181		0.722	0.729
DRB5*0101	343	0.654	0.652	0.646

Average			0.732	0.747
Average I		0.736	0.738	0.739
Average II			0.711	0.776

"Average" is the average over 14 alleles. "Average I" is the average over 11 alleles predictable by TEPITOPE. "Average II" is the average over 3 alleles not predictable by TEPITOPE. We obtain the PSSMs of TEPITOPE and TEPITOPEpan from their public servers ProPred (http://www.imtech.res.in/raghava/propred/page4.html) and TEPITOPEpan (http://www.biokdd.fudan.edu.cn/Service/TEPITOPEpan/TEPITOPEpan.html), respectively.

### Performance on the MHCBench dataset

In order to evaluate the performance of OWA-PSSM compared with TEPITOPE, MultiRTA, NetMHCI-Ipan2.0 and TEPITOPEpan, eight datasets consisting of binders and non-binders for DRB1*0401 were retrieved from the MHCBench database. As shown in Table 4 the performance of OWA-PSSM is similar to those of TEPITOPE and NetMHCIIpan2.0. It can also be observed that OWA-PSSM performs best in all eight datasets, and significantly outperforms TEPITOPEpan (*p <*0.01, paired t-test) and MultiRTA (*p <*0.01, paired t-test).

**Table 4:** The performance on the MHCBench dataset in terms of AUC.

Dataset	# peptides	OWA-PSSM	TEPITOPE	TEPITOPEpan	MultiRTA	NetMHCIIpan-2.0
Set 1	1017	0.768	0.766	0.764	0.713	0.765
Set 2	673	0.735	0.734	0.727	0.685	0.739
Set 3a	590	0.739	0.736	0.734	0.701	0.710
Set 3b	495	0.756	0.753	0.748	0.715	0.753
Set 4a	646	0.753	0.750	0.748	0.699	0.759
Set 4b	584	0.746	0.745	0.738	0.706	0.751
Set 5a	117	0.655	0.668	0.640	0.599	0.649
Set 5b	85	0.664	0.689	0.646	0.597	0.640

Average		0.727	0.730	0.718	0.677	0.721

The PSSMs of TEPITOPE, TEPITOPEpan and the results of MultiRTA were obtained from their respective web servers. The prediction of NetMHCIIpan-2.0 were computed by its stand-alone software package.

### Performance on the HLA-DR ligand dataset

Here, we evaluate the OWA-PSSM method on a large-scale MHC II ligand dataset. This dataset covers 1164 HLA-DR ligands restricted to 28 HLA-DR alleles [5,14]. We applied a similar approach as NetMHCIIpan-2.0, in which each ligand source protein was divided into overlapping k-mers with lengths equal to the annotated ligand, and all k-mers without the annotated ligand were labeled as negatives. The results are shown in Table 5. The prediction performance of OWA-PSSM is significantly better than that of TEPITOPEpan (*p <*0.05, paired t-test). Specifically, the OWA-PSSM method outperforms TEPITOPEpan in 16 out of 28 alleles. Comparing the prediction accuracy of OWA-PSSM with those of MultiRTA and NetMHCIIpan2.0 on this ligand dataset, we find that their AUC score distributions are not significantly different (*p >*0.05, paired t-test). For the 17 alleles predictable by TEPITOPE, TEPITOPE performs best for 11 alleles and OWA-PSSM performs best for 6 alleles. However, these differences are not statistically significant (*p >*0.05, paired t-test).

**Table 5:** Prediction performance on the HLA-DR ligand dataset.

Allele	# ligands	OWA-PSSM	TEPITOPE	TEPITOPEpan	MultiRTA	NetMHCIIpan-2.0
DRB1*0101	53	0.827	0.833	0.834	0.833	0.835
DRB1*0102	5	0.889	0.895	0.892	0.935	0.927
DRB1*0301	88	0.667	0.673	0.671	0.652	0.789
DRB1*0401	468	0.831	0.833	0.826	0.771	0.875
DRB1*0402	36	0.882	0.885	0.880	0.768	0.667
DRB1*0403	1	0.954		0.954	1.000	0.845
DRB1*0404	42	0.779	0.775	0.797	0.711	0.765
DRB1*0405	36	0.804	0.809	0.778	0.729	0.856
DRB1*0701	47	0.698	0.697	0.696	0.720	0.744
DRB1*0801	39	0.694	0.697	0.656	0.541	0.643
DRB1*0802	1	0.930	0.916	0.923	0.532	0.978
DRB1*0803	1	0.229		0.149	0.383	0.292
DRB1*0901	6	0.750		0.659	0.842	0.957
DRB1*1001	183	0.783		0.770	0.827	0.866
DRB1*1101	35	0.828	0.835	0.831	0.838	0.896
DRB1*1104	8	0.868	0.870	0.856	0.811	0.911
DRB1*1201	11	0.801		0.828	0.847	0.863
DRB1*1301	16	0.819	0.824	0.813	0.745	0.724
DRB1*1302	19	0.743	0.742	0.735	0.720	0.561
DRB1*1401	9	0.712		0.730	0.704	0.810
DRB1*1501	22	0.720	0.718	0.717	0.663	0.671
DRB1*1502	3	0.773	0.767	0.774	0.706	0.665
DRB1*1601	2	0.621		0.630	0.918	0.849
DRB3*0101	2	0.888		0.918	0.953	0.971
DRB3*0301	5	0.907		0.783	0.939	0.948
DRB4*0101	6	0.500		0.491	0.515	0.726
DRB4*0103	2	0.941		0.753	0.745	0.827
DRB5*0101	18	0.823	0.842	0.835	0.777	0.847

Average	1164	0.774		0.756	0.754	0.797
Average I		0.799	0.801	0.795	0.732	0.786
Average II		0.735		0.697	0.789	0.814

"Average" is the average over 28 alleles. "Average I" is the average over 17 alleles predictable by TEPITOPE.

"Average II" is the average over 11 alleles not predictable by TEPITOPE. The PSSMs of TEPITOPE, TEPITOPEpan and the results of MultiRTA were obtained from their respective web servers. The prediction of NetMHCIIpan-2.0 were obtained directly from its publication.

### Performance on the HLA-DR T cell epitope dataset

A T cell epitope is an antigen fragment that is recognized by T cells. Identifying T cell epitopes is vital for vaccine design. A large-scale T-cell epitopes dataset used in [14] is tested here. The prediction performance is showed in Table 6. The OWA-PSSM method performs significantly better than MultiRTA (*p <*0.01, paired t-test) in identifying T cell epitopes. Comparing the prediction accuracy of OWA-PSSM with those of TEPITOPEpan and NetMHCIIpan2.0 on this T cell epitope dataset, we find that their AUC score distributions are not significantly different (*p >*0.05, paired t-test). TEPITOPE is one of the best approaches in identifying T cell epitopes. Compared with this approach, OWA-PSSM performs best for 12 of the 20 alleles predictable by TEPITOPE, while TEPITOPE performs best on 8 alleles (the difference is not significant, *p >*0.05, paired t-test).

**Table 6:** Prediction performance on the HLA-DR T-cell epitope dataset.

Allele	# epitopes	OWA-PSSM	TEPITOPE	TEPITOPEpan	MultiRTA	NetMHCIIpan-2.0
DRB1*0101	125	0.807	0.795	0.808	0.786	0.810
DRB1*0102	4	0.790	0.761	0.792	0.822	0.879
DRB1*0103	5	0.837		0.719	0.528	0.667
DRB1*0301	173	0.632	0.637	0.640	0.655	0.683
DRB1*0401	342	0.747	0.741	0.743	0.707	0.775
DRB1*0402	33	0.575	0.574	0.571	0.521	0.570
DRB1*0403	14	0.904		0.905	0.848	0.896
DRB1*0404	46	0.732	0.732	0.738	0.715	0.744
DRB1*0405	21	0.745	0.746	0.722	0.579	0.626
DRB1*0406	6	0.766		0.753	0.869	0.741
DRB1*0407	4	0.808		0.824	0.749	0.668
DRB1*0408	2	0.930	0.930	0.930	0.999	0.986
DRB1*0701	56	0.737	0.720	0.736	0.736	0.742
DRB1*0703	1	0.905	0.911	0.915	0.707	0.896
DRB1*0801	4	0.586	0.554	0.640	0.716	0.663
DRB1*0802	2	0.848	0.866	0.850	0.685	0.754
DRB1*0803	2	0.548		0.516	0.707	0.852
DRB1*0901	13	0.729		0.697	0.636	0.738
DRB1*1001	4	0.870		0.835	0.789	0.875
DRB1*1101	88	0.752	0.745	0.751	0.703	0.815
DRB1*1102	1	0.843	0.828	0.822	0.503	0.493
DRB1*1103	3	0.333		0.328	0.480	0.510
DRB1*1104	6	0.793	0.810	0.805	0.666	0.807
DRB1*1201	3	0.876		0.887	0.862	0.970
DRB1*1301	15	0.767	0.783	0.756	0.642	0.632
DRB1*1302	10	0.832	0.809	0.813	0.781	0.860
DRB1*1303	3	0.482		0.562	0.515	0.604
DRB1*1401	16	0.718		0.781	0.697	0.789
DRB1*1404	1	0.930		0.949	0.938	0.956
DRB1*1405	2	0.861		0.807	0.848	0.839
DRB1*1501	193	0.688	0.681	0.690	0.665	0.722
DRB1*1502	20	0.611	0.605	0.608	0.570	0.681
DRB1*1503	2	0.802		0.829	0.531	0.874
DRB1*1601	5	0.684		0.699	0.721	0.724
DRB1*1602	3	0.885		0.912	0.886	0.984
DRB3*0101	12	0.875		0.833	0.883	0.895
DRB3*0202	10	0.588		0.613	0.466	0.539
DRB3*0301	1	0.988		0.885	0.906	0.966
DRB4*0101	17	0.663		0.560	0.583	0.789
DRB4*0103	1	0.990		0.990	0.992	0.991
DRB5*0101	55	0.746	0.738	0.747	0.752	0.802
DRB5*0102	1	0.909		0.870	0.752	0.987

Average		0.764	0.748	0.758	0.717	0.781
Average I		0.753	0.748	0.754	0.696	0.747
Average II		0.775		0.761	0.736	0.812

"Average" is the average over 42 alleles. "Average I" is the average over 20 alleles predictable by TEPITOPE.

"Average II" is the average over 22 alleles not predictable by TEPITOPE. The PSSMs of TEPITOPE, TEPITOPEpan and the results of MultiRTA were obtained from their respective web servers. The prediction of NetMHCIIpan-2.0 were obtained directly from its publication.

### Identification of the peptide binding cores

The lengths of the peptides which bind to MHC II range from 9 to 25 amino acids. However, only a segment of the peptide plays a significant role in binding to a MHC II molecule which is referred to as a binding core. Identifying the binding core correctly is very important for the MHC II/peptide binding study. A dataset containing 41 X-ray crystallographic structures is employed to evaluate the prediction of OWA-PSSM in identifying binding cores. As showed in Table 7, OWA-PSSM is the only method that correctly identifies the binding cores for all complexes. TEPITOPEpan, MultiRTA and NetMHCIIpan2.0 misidentify 2, 5 and 9 complexes, respectively. For the 39 MHC II/peptide complexes whose MHC II molecules are predictable by TEPITOPE, both TEPITOPE and OWA-PSSM can correctly predict the binding cores. However, OWAPSSM is able to identify the binding cores of all 41 complexes correctly regardless of whether the MHC II alleles are predictable by TEPITOPE or not.

**Table 7:** Comparison of OWA-PSSM with four pan-specific methods in identifying MHC II-peptide binding cores.

PDB ID	OWA-PSSM	TEPITOPE	TEPITOPEpan	MultiRTA	NetMHCIIpan-2.0
4E41	IGILNAAKV	IGILNAAKV	IGILNAAKV	IGILNAAKV	**LIGILNAAK**
1A6A	MRMATPLLM	MRMATPLLM	MRMATPLLM	MRMATPLLM	MRMATPLLM
1AQD	WRFLRGYHQ	WRFLRGYHQ	WRFLRGYHQ	WRFLRGYHQ	WRFLRGYHQ
1BX2	VHFFKNIVT	VHFFKNIVT	VHFFKNIVT	VHFFKNIVT	**VVHFFKNIV**
1DLH	YVKQNTLKL	YVKQNTLKL	YVKQNTLKL	YVKQNTLKL	YVKQNTLKL
1FV1	FKNIVTPRT	FKNIVTPRT	FKNIVTPRT	**VHFFKNIVT**	**FFKNIVTPR**
1FYT	YVKQNTLKL	YVKQNTLKL	YVKQNTLKL	YVKQNTLKL	YVKQNTLKL
1H15	YHFVKKHVH	YHFVKKHVH	YHFVKKHVH	YHFVKKHVH	YHFVKKHVH
1HQR	FKNIVTPRT	FKNIVTPRT	FKNIVTPRT	**VHFFKNIVT**	**FFKNIVTPR**
1HXY	YVKQNTLKL	YVKQNTLKL	YVKQNTLKL	YVKQNTLKL	YVKQNTLKL
1J8H	YVKQNTLKL	YVKQNTLKL	YVKQNTLKL	YVKQNTLKL	YVKQNTLKL
1JWM	YVKQNTLKL	YVKQNTLKL	YVKQNTLKL	YVKQNTLKL	YVKQNTLKL
1JWS	YVKQNTLKL	YVKQNTLKL	YVKQNTLKL	YVKQNTLKL	YVKQNTLKL
1JWU	YVKQNTLKL	YVKQNTLKL	YVKQNTLKL	YVKQNTLKL	YVKQNTLKL
1KG0	YVKQNTLKL	YVKQNTLKL	YVKQNTLKL	YVKQNTLKL	YVKQNTLKL
1KLG	IGILNAAKV	IGILNAAKV	IGILNAAKV	IGILNAAKV	**LIGILNAAK**
1KLU	IGTLNAAKV	IGTLNAAKV	IGTLNAAKV	IGTLNAAKV	IGTLNAAKV
1LO5	YVKQNTLKL	YVKQNTLKL	YVKQNTLKL	YVKQNTLKL	YVKQNTLKL
1PYW	FVKQNAAAL	FVKQNAAAL	FVKQNAAAL	FVKQNAAAL	FVKQNAAAL
1R5I	YVKQNTLKL	YVKQNTLKL	YVKQNTLKL	YVKQNTLKL	YVKQNTLKL
1SJE	VIPMFSALS	VIPMFSALS	VIPMFSALS	VIPMFSALS	VIPMFSALS
1SJH	VIPMFSALS	VIPMFSALS	VIPMFSALS	VIPMFSALS	VIPMFSALS
1T5W	YSDQATPLL	YSDQATPLL	YSDQATPLL	**SDQATPLLL**	YSDQATPLL
1T5X	YSDQATPLL	YSDQATPLL	YSDQATPLL	**SDQATPLLL**	YSDQATPLL
1YMM	VHFFKNIVT	VHFFKNIVT	VHFFKNIVT	VHFFKNIVT	VHFFKNIVT
1ZGL	FKNIVTPRT	FKNIVTPRT	FKNIVTPRT	**VHFFKNIVT**	**FFKNIVTPR**
2FSE	FKGEQGPKG	FKGEQGPKG	FKGEQGPKG	FKGEQGPKG	FKGEQGPKG
2G9H	YVKQNTLKL	YVKQNTLKL	YVKQNTLKL	YVKQNTLKL	YVKQNTLKL
2IAM	IGILNAAKV	IGILNAAKV	IGILNAAKV	IGILNAAKV	**LIGILNAAK**
2IAN	IGTLNAAKV	IGTLNAAKV	IGTLNAAKV	IGTLNAAKV	IGTLNAAKV
2ICW	YVKQNTLKL	YVKQNTLKL	YVKQNTLKL	YVKQNTLKL	YVKQNTLKL
2IPK	WVKQNTLKL	WVKQNTLKL	WVKQNTLKL	WVKQNTLKL	WVKQNTLKL
2OJE	YVKQNTLKL	YVKQNTLKL	YVKQNTLKL	YVKQNTLKL	YVKQNTLKL
2Q6W	WRSDEALPL	-	WRSDEALPL	WRSDEALPL	WRSDEALPL
2SEB	MRADAAAGG	MRADAAAGG	**YMRADAAAG**	MRADAAAGG	**YMRADAAAG**
3C5J	IILNHPGQI	-	**VIILNHPGQ**	IILNHPGQI	IILNHPGQI
3L6F	YEKLSAEQS	YEKLSAEQS	YEKLSAEQS	YEKLSAEQS	YEKLSAEQS
3PDO	MRMATPLLM	MRMATPLLM	MRMATPLLM	MRMATPLLM	MRMATPLLM
3PGD	MRMATPLLM	MRMATPLLM	MRMATPLLM	MRMATPLLM	**KMRMATPLL**
3S4S	YVKQNTLKL	YVKQNTLKL	YVKQNTLKL	YVKQNTLKL	YVKQNTLKL
3S5L	YVKQNTLKL	YVKQNTLKL	YVKQNTLKL	YVKQNTLKL	YVKQNTLKL

Correct	41/41		39/41	36/41	32/41
Correct I	39/39	39/39	38/39	34/39	30/39
Correct II	2/2		1/2	2/2	2/2

Incorrectly predicted binding cores are highlighted in bold. "Correct" gives the number of correctly identified cores over 41 X-ray structures. "Correct I" gives the number of correctly identified cores over 39 X-ray structures whose DRB alleles are predictable by TEPITOPE. "Correct II" gives the number of correctly identified cores over 2 X-ray structures whose DRB alleles are not predictable by TEPITOPE. The PSSMs of TEPITOPE, TEPITOPEpan and the results of MultiRTA, NetMHCIIpan-2.0 were obtained from their respective web servers.

## Discussion and conclusion

The PSSM based approaches have been demonstrated to be a powerful technique for MHC/peptide binding prediction [26,33,34]. A PSSM is a motif matrix which can succinctly describe a MHC/peptide binding motif. The TEPITOPE method is the best known PSSM based pan-specific approach for MHC II binding prediction. It determines 35 distinct pocket profiles in vitro based on 11 HLA-DR alleles. Although TEPITOPE can solely perform prediction for 50 HLA-DR alleles, it has been shown to be one of the best performing approaches in HLA-DR ligand/epitope prediction. Furthermore, it is the best method in identifying HLA-DR binging cores. The TEPITOPEpan method is also a PSSM based method whose PSSMs are generated based on those 35 pocket profiles determined by TEPITOPE. TEPITOPEpan uses the same approach as PickPocket to compute the similarity score between two pocket pseudo-sequences and the weights over pocket profiles by using BLOSUM62. Based on this approach, the similarity score is likely to be negative, and while setting the negative score to zero, it is probable that the similarity scores among all pocket profiles are zero, and the weights cannot be computed. On the other hand, OWA-PSSM does not encounter this problem since its similarity scores and weights associated with pocket profiles are positive.

The performance of OWA-PSSM and TEPITOPE is similar for the alleles predictable by TEPITOPE. While OWA-PSSM can make prediction for a much higher number of HLA-DR molecules then TEPITOPE. In addition, the method is extensively evaluated on five benchmark datasets, and is shown to be the best approach in identifying binding cores compared with four state-of-the-art or recently proposed pan-specific MHC II prediction approaches, TEPITOPE, MultiRTA, NetMHCIIpan2.0 and TEPITOPEpan. Additionally, the method performs comparably to TEPITOPE and NetMHCIIpan2.0 in identifying HLA-DR epitopes and ligands, and it significantly outperforms TEPITOPEpan in identifying HLA-DR ligands and MultiRTA in the identification of HLA-DR T cell epitopes.

Here, we develop a new MHC II binding prediction approach, which we call OWA-PSSM. This method is a significantly extended version of the TEPITOPE method. In particular, we preserve the advantage of TEPITOPE. Positions 1, 4, 6, 7 and 9 in a binding core of a MHC II molecule are estimated to be anchor positions which determine the peptide binding affenity. Identifying the P1 position of the binding core is an essential step for MHC II ligand/epitope prediction, and the TEPITOPE method clearly reveals the MHC II molecules' preferred amino acids at the P1 position. As a result, OWA-PSSM is also successful in predicting MHC II/ligands, epitopes and binding cores by using a similar approach. For pocket 4, 6, 7 and 9, the MHC II molecules are highly polymorphic and the pseudo sequences in the same pocket are highly diverse, hence the TEPITOPE method is unable to predict those DRB alleles with pseudo sequences different from its original 35 pseudo sequences. In this work, through introducing a new weighting scheme that is inspired by the OWA operator, we can make prediction for up to 879 MHC II molecules. In addition, this method is fast and robust in identifying HLA-DR ligands, epitopes and binding cores.

## Competing interests

The authors declare that they have no competing interests.

## Authors' contributions

WJS formulated the algorithm and performed the experiments. SZ designed the experimental protocols.

HSW initiated the project and designed the overall algorithmic framework. All authors read and approved the manuscript.

## Declarations

The publication costs for this article were funded by the corresponding author.

This article has been published as part of *Proteome Science *Volume 11 Supplement 1, 2013: Selected articles from the IEEE International Conference on Bioinformatics and Biomedicine 2012: Proteome Science. The full contents of the supplement are available online at http://www.proteomesci.com/supplements/11/S1.
